# Diaqua­bis­(dimethyl sulfoxide-κ*O*)bis(saccharinato-κ*N*)cobalt(II)

**DOI:** 10.1107/S1600536811045296

**Published:** 2011-11-05

**Authors:** Fezile S. W. Potwana, Werner E. Van Zyl

**Affiliations:** a School of Chemistry, University of KwaZulu-Natal, Westville Campus, Private Bag X54001, Durban 4000, South Africa

## Abstract

The title complex, [Co(C_7_H_4_NO_3_S)_2_(C_2_H_6_OS)_2_(H_2_O)_2_], contains a Co^2+^ cation in an octa­hedral coordination environment. The metal atom is surrounded by two different neutral ligands, namely dimethyl­sulfoxide (DMSO) and water, each coordinating through the O atom. The anionic saccharinate (sac; 1,1,3-trioxo-2,3-dihydro-1λ^6^,2-benzothia­zol-2-ide) ligand coordinates through the N atom. Each of the three similar ligand pairs is in a *trans* configuration with respect to each other. The Co atom lies on a crystallographic center of symmetry and the octa­hedral geometry is not significantly distorted. A short O—H⋯O hydrogen bond is present between a water H atom and the ketone O atom; two longer hydrogen bonds (intra- and inter­molecular) are also present between a water H and a sulfonic O atom, forming a supramolecular assembly through head-to-tail aggregation between adjacent complexes.

## Related literature

For a general review article on the coordination chemistry of saccharinate ligands, see: Baran & Yilmaz (2006 ▶). For cobalt(II) saccharinate complexes, see: Deng *et al.* (2008 ▶) and for cobalt(II) complexes with saccharinate as a non-coordinating ligand, see: Batsanov *et al.* (2011 ▶). For the preparation of cobalt(II) and other divalent metal precursor complexes, see: Haider *et al.* (1985 ▶).
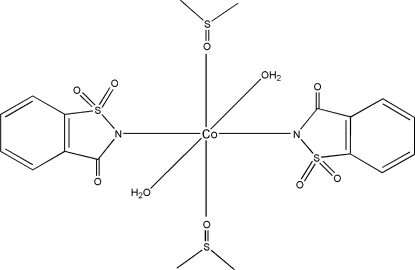


## Experimental

### 

#### Crystal data

[Co(C_7_H_4_NO_3_S)_2_(C_2_H_6_OS)_2_(H_2_O)_2_]
*M* *_r_* = 615.56
Monoclinic, 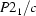

*a* = 10.2304 (3) Å
*b* = 15.1418 (6) Å
*c* = 7.8615 (3) Å
β = 98.068 (2)°
*V* = 1205.75 (8) Å^3^
*Z* = 2
Mo *K*α radiation
μ = 1.12 mm^−1^
*T* = 173 K
0.27 × 0.15 × 0.10 mm

#### Data collection

Nonius KappaCCD diffractometer
Absorption correction: multi-scan (*SADABS*; Bruker, 2001 ▶) *T*_min_ = 0.753, *T*_max_ = 0.897
5888 measured reflections
3000 independent reflections
2301 reflections with *I* > 2σ(*I*)
*R*_int_ = 0.018

#### Refinement

*R*[*F* ^2^ > 2σ(*F* ^2^)] = 0.030
*wR*(*F* ^2^) = 0.081
*S* = 1.06
3000 reflections
171 parameters
2 restraints
H atoms treated by a mixture of independent and constrained refinement
Δρ_max_ = 0.71 e Å^−3^
Δρ_min_ = −0.49 e Å^−3^

### 

Data collection: *COLLECT* (Nonius, 1998 ▶); cell refinement: *DENZO-SMN* (Otwinowski & Minor, 1997 ▶); data reduction: *DENZO-SMN*; program(s) used to solve structure: *SHELXS97* (Sheldrick, 2008 ▶); program(s) used to refine structure: *SHELXL97* (Sheldrick, 2008 ▶); molecular graphics: *SHELXTL* (Sheldrick, 2008 ▶); software used to prepare material for publication: *SHELXL97*.

## Supplementary Material

Crystal structure: contains datablock(s) I, global. DOI: 10.1107/S1600536811045296/br2180sup1.cif

Structure factors: contains datablock(s) I. DOI: 10.1107/S1600536811045296/br2180Isup2.hkl

Additional supplementary materials:  crystallographic information; 3D view; checkCIF report

## Figures and Tables

**Table 1:** Hydrogen-bond geometry (Å, °)

*D*—H⋯*A*	*D*—H	H⋯*A*	*D*⋯*A*	*D*—H⋯*A*
O5—H5*A*⋯O3^i^	0.98	1.73	2.646 (2)	155 (3)
O5—H5*B*⋯O1	0.98	2.09	2.803 (2)	128 (2)
O5—H5*B*⋯O1^ii^	0.98	2.06	2.904 (2)	143 (2)
C8—H8*B*⋯O5^iii^	0.98	2.54	3.277 (2)	132
C8—H8*B*⋯O4^iv^	0.98	2.51	3.383 (2)	148
C9—H9*A*⋯O3^i^	0.98	2.58	3.363 (2)	137
C9—H9*B*⋯O4^iv^	0.98	2.58	3.432 (2)	146

Symmetry codes: (i) 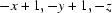; (ii) 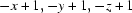; (iii) 
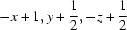
; (iv) 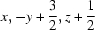.

**Fig. 1.**
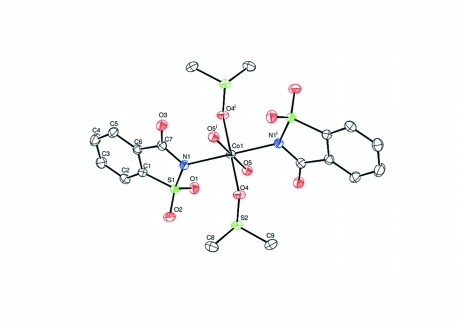
The ORTEP molecular structure of the title complex, shown with 50% probability ellipsoids.

## supplementary crystallographic information

### Comment

Saccharin (*o*-sulfobenzimide; 1,2-benzothiazole-3(2*H*)-one 1,1-dioxide; Hsac) is a widely used artificial sweetening agent. The imino hydrogen is acidic and can be readily deprotonated. The coordination chemistry of this anion is versatile due to the different coordination sites to metallic centers it can accommodate, *i.e.*, one N, one O (carbonylic) and two O (sulfonic) atoms. These donor atoms of the anion can thus readily generate either N– or O-monodentate or bidentate (N, O) coordination. Saccharin is normally used as the sodium or calcium salt which dramatically improves water solubility. Most metal complexes contain the deprotonated form of saccharin, and this saccharinate anion (sac) is commercially available as the sodium salt, used in the present study. The reaction of sodium saccharinate with the first row divalent transition metal ions results in coordination complexes with general formula [*M*(sac)_2_(H_2_O)_4_].2H_2_O, (*M* = V, Cr, Mn, Fe, Co, Ni, Cu, Zn), which all show a clear preference to bind through the deprotonated anionic N-atom (Baran and Yilmaz, 2006). These octahedral complexes contain two N-bonded sac ligands in *trans* positions, and complexes of the type [*M*(sac)_2_(H_2_O)_4_].2H_2_O are thus commonly used as precursors in the synthesis of mixed-ligand saccharinate complexes. The aqua ligands in these metal complexes are labile and readily displaced by direct reaction of neutral ligands. The addition of the ligands to the solutions of the complexes usually results in the substitution of all four aqua ligands, thereby forming stable new mixed-ligand complexes. In cases where the incoming neutral ligand is relatively bulky, as in the present study, it causes steric hindrance and only two of the four aqua ligands become displaced in order for the Co center to remain octahedral. Although there are a number of Co(II) saccharinate complexes previously reported (Batsanov *et al.*, 2011, and refs. therein), the present study reports the first example of a structurally characterized Co(II) complex that contains both saccharinate and dmso ligands.

### Experimental

[Co(sac)_2_(H_2_O)_4_].2H_2_O was prepared as per literature method (Haider *et al.*, 1985). The red crystals of [Co(sac)_2_(H_2_O)_4_].2H_2_O (0.932 g; 1.80 mmol) was placed in a 100 ml beaker and dissolved in excess amount of dimethyl sulfoxide (dmso) (20 ml). The reaction mixture was gently heated on a heating plate with stirring to reduce the volume of dmso to ~7 ml. The beaker was removed from the heat source and allowed to stand for 10 days during which time large light red blocky crystals of the title compound were obtained. Yield (1.00 g, 93.0%). Mp: 393 K; 120 ° C. IR-ATR (cm^-1^): 3486.98, 3005.41 n(OH); 1618 n(C=O); 1584, 1460 n(C=C); 1256 n(O=S=O); 1141, 949 n(S=O). No NMR data were recorded due to the paramagnetic nature of the Co(II) complex. Single crystals were obtained by slow evaporation of dmso solvent.

### Refinement

All hydrogen atoms could be found in the difference electron density maps. All, except H5A and H5B on O5, were placed in idealised positions refining in riding models with Uiso set at 1.2 or 1.5 times those of their parent atoms. The water hydrogen atoms H5A and H5B were located in the difference electron density maps and refined with independent isotropic temperature factors and simple bond length constraints of d(O-H) = 0.980 (2) Å.

### Crystal data

**Table:**

[Co(C_7_H_4_NO_3_S)_2_(C_2_H_6_OS)_2_(H_2_O)_2_]	*F*(000) = 634
*M**_r_* = 615.56	*D*_x_ = 1.695 Mg m^−^^3^
Monoclinic, *P*2_1_/*c*	Mo *K*α radiation, λ = 0.71073 Å
Hall symbol: -P 2ybc	Cell parameters from 5888 reflections
*a* = 10.2304 (3) Å	θ = 2.4–28.3°
*b* = 15.1418 (6) Å	µ = 1.12 mm^−^^1^
*c* = 7.8615 (3) Å	*T* = 173 K
β = 98.068 (2)°	Block, light red
*V* = 1205.75 (8) Å^3^	0.27 × 0.15 × 0.10 mm
*Z* = 2	

### Data collection

**Table:**

Nonius KappaCCD diffractometer	3000 independent reflections
Radiation source: fine-focus sealed tube	2301 reflections with *I* > 2σ(*I*)
graphite	*R*_int_ = 0.018
1.2° φ scans and ω scans	θ_max_ = 28.3°, θ_min_ = 2.4°
Absorption correction: multi-scan (*SADABS*; Bruker, 2001)	*h* = −13→13
*T*_min_ = 0.753, *T*_max_ = 0.897	*k* = −20→20
5888 measured reflections	*l* = −10→10

### Refinement

**Table:**

Refinement on *F*^2^	Secondary atom site location: difference Fourier map
Least-squares matrix: full	Hydrogen site location: inferred from neighbouring sites
*R*[*F*^2^ > 2σ(*F*^2^)] = 0.030	H atoms treated by a mixture of independent and constrained refinement
*wR*(*F*^2^) = 0.081	*w* = 1/[σ^2^(*F*_o_^2^) + (0.0497*P*)^2^] where *P* = (*F*_o_^2^ + 2*F*_c_^2^)/3
*S* = 1.06	(Δ/σ)_max_ < 0.001
3000 reflections	Δρ_max_ = 0.71 e Å^−^^3^
171 parameters	Δρ_min_ = −0.49 e Å^−^^3^
2 restraints	Extinction correction: *SHELXL97* (Sheldrick, 2008), Fc^*^=kFc[1+0.001xFc^2^λ^3^/sin(2θ)]^-1/4^
Primary atom site location: structure-invariant direct methods	Extinction coefficient: 0.0082 (11)

### Special details

**Table:**

Geometry. All e.s.d.'s (except the e.s.d. in the dihedral angle between two l.s. planes) are estimated using the full covariance matrix. The cell e.s.d.'s are taken into account individually in the estimation of e.s.d.'s in distances, angles and torsion angles; correlations between e.s.d.'s in cell parameters are only used when they are defined by crystal symmetry. An approximate (isotropic) treatment of cell e.s.d.'s is used for estimating e.s.d.'s involving l.s. planes.
Refinement. Refinement of *F*^2^ against ALL reflections. The weighted *R*-factor *wR* and goodness of fit *S* are based on *F*^2^, conventional *R*-factors *R* are based on *F*, with *F* set to zero for negative *F*^2^. The threshold expression of *F*^2^ > σ(*F*^2^) is used only for calculating *R*-factors(gt) *etc*. and is not relevant to the choice of reflections for refinement. *R*-factors based on *F*^2^ are statistically about twice as large as those based on *F*, and *R*- factors based on ALL data will be even larger.

### Fractional atomic coordinates and isotropic or equivalent isotropic displacement parameters (Å^2^)

**Table:**

	*x*	*y*	*z*	*U*_iso_*/*U*_eq_	
Co1	0.5000	0.5000	0.0000	0.01579 (11)	
S1	0.27267 (4)	0.50687 (3)	0.28359 (5)	0.01976 (12)	
S2	0.51966 (4)	0.68690 (3)	0.19988 (6)	0.02130 (13)	
O1	0.37735 (12)	0.46878 (9)	0.40314 (15)	0.0278 (3)	
O2	0.24179 (13)	0.59763 (9)	0.31675 (17)	0.0305 (3)	
O3	0.18849 (12)	0.43540 (8)	−0.16410 (15)	0.0251 (3)	
O4	0.51937 (12)	0.63688 (8)	0.03114 (15)	0.0222 (3)	
O5	0.60377 (12)	0.47674 (8)	0.24012 (16)	0.0218 (3)	
H5A	0.6902 (13)	0.5053 (17)	0.246 (4)	0.087 (11)*	
H5B	0.570 (3)	0.4869 (16)	0.3492 (18)	0.062 (8)*	
N1	0.30080 (14)	0.49352 (9)	0.08660 (19)	0.0193 (3)	
C1	0.13015 (16)	0.44084 (11)	0.2695 (2)	0.0192 (4)	
C2	0.05764 (17)	0.41415 (12)	0.3962 (2)	0.0240 (4)	
H2	0.0820	0.4307	0.5129	0.029*	
C3	−0.05215 (19)	0.36209 (12)	0.3448 (3)	0.0296 (4)	
H3	−0.1056	0.3433	0.4275	0.036*	
C4	−0.08537 (19)	0.33692 (14)	0.1742 (3)	0.0324 (5)	
H4	−0.1611	0.3010	0.1428	0.039*	
C5	−0.01053 (17)	0.36300 (12)	0.0487 (2)	0.0259 (4)	
H5	−0.0334	0.3452	−0.0676	0.031*	
C6	0.09874 (16)	0.41590 (11)	0.0990 (2)	0.0196 (4)	
C7	0.19847 (16)	0.44925 (11)	−0.0076 (2)	0.0192 (4)	
C8	0.39348 (18)	0.76726 (13)	0.1549 (2)	0.0296 (4)	
H8A	0.4055	0.7992	0.0498	0.044*	
H8B	0.3981	0.8089	0.2509	0.044*	
H8C	0.3072	0.7380	0.1391	0.044*	
C9	0.65935 (19)	0.75778 (13)	0.2148 (3)	0.0348 (5)	
H9A	0.7402	0.7223	0.2331	0.052*	
H9B	0.6581	0.7985	0.3114	0.052*	
H9C	0.6567	0.7915	0.1080	0.052*	

### Atomic displacement parameters (Å^2^)

**Table:**

	*U*^11^	*U*^22^	*U*^33^	*U*^12^	*U*^13^	*U*^23^
Co1	0.01863 (18)	0.01480 (18)	0.01393 (18)	−0.00075 (12)	0.00224 (13)	−0.00099 (12)
S1	0.0190 (2)	0.0247 (2)	0.0160 (2)	−0.00384 (16)	0.00399 (17)	−0.00383 (16)
S2	0.0269 (2)	0.0180 (2)	0.0189 (2)	−0.00124 (17)	0.00312 (17)	−0.00226 (17)
O1	0.0216 (7)	0.0433 (8)	0.0182 (7)	−0.0027 (6)	0.0013 (5)	−0.0020 (6)
O2	0.0354 (8)	0.0253 (7)	0.0324 (8)	−0.0053 (6)	0.0103 (6)	−0.0102 (6)
O3	0.0246 (6)	0.0333 (7)	0.0172 (6)	−0.0048 (5)	0.0023 (5)	−0.0029 (5)
O4	0.0319 (7)	0.0156 (6)	0.0195 (6)	−0.0010 (5)	0.0049 (5)	−0.0023 (5)
O5	0.0237 (7)	0.0248 (6)	0.0169 (6)	−0.0007 (5)	0.0034 (5)	0.0001 (5)
N1	0.0181 (7)	0.0241 (8)	0.0162 (7)	−0.0029 (6)	0.0042 (6)	−0.0019 (6)
C1	0.0188 (8)	0.0185 (8)	0.0202 (9)	0.0005 (7)	0.0026 (7)	−0.0017 (7)
C2	0.0255 (9)	0.0261 (9)	0.0216 (9)	0.0020 (8)	0.0077 (8)	−0.0009 (8)
C3	0.0288 (10)	0.0325 (11)	0.0301 (11)	−0.0060 (8)	0.0129 (8)	0.0005 (8)
C4	0.0263 (10)	0.0361 (11)	0.0351 (11)	−0.0113 (9)	0.0057 (8)	−0.0021 (9)
C5	0.0231 (9)	0.0301 (11)	0.0237 (9)	−0.0043 (7)	0.0005 (8)	−0.0050 (8)
C6	0.0180 (8)	0.0205 (9)	0.0200 (9)	0.0017 (7)	0.0019 (7)	0.0004 (7)
C7	0.0189 (8)	0.0209 (9)	0.0176 (9)	0.0022 (7)	0.0022 (7)	0.0011 (7)
C8	0.0351 (11)	0.0248 (10)	0.0291 (11)	0.0070 (8)	0.0049 (9)	−0.0041 (8)
C9	0.0332 (11)	0.0313 (11)	0.0399 (12)	−0.0098 (9)	0.0058 (9)	−0.0143 (9)

### Geometric parameters (Å, °)

**Table:**

Co1—O5^i^	2.0620 (12)	C1—C2	1.383 (2)
Co1—O5	2.0620 (12)	C1—C6	1.386 (2)
Co1—O4^i^	2.0932 (12)	C2—C3	1.385 (3)
Co1—O4	2.0932 (12)	C2—H2	0.9500
Co1—N1^i^	2.2396 (14)	C3—C4	1.390 (3)
Co1—N1	2.2396 (14)	C3—H3	0.9500
S1—O2	1.4420 (14)	C4—C5	1.389 (3)
S1—O1	1.4423 (13)	C4—H4	0.9500
S1—N1	1.6270 (15)	C5—C6	1.387 (2)
S1—C1	1.7586 (17)	C5—H5	0.9500
S2—O4	1.5272 (12)	C6—C7	1.496 (2)
S2—C8	1.7731 (18)	C8—H8A	0.9800
S2—C9	1.7779 (18)	C8—H8B	0.9800
O3—C7	1.2383 (19)	C8—H8C	0.9800
O5—H5A	0.980 (2)	C9—H9A	0.9800
O5—H5B	0.980 (2)	C9—H9B	0.9800
N1—C7	1.370 (2)	C9—H9C	0.9800
			
O5^i^—Co1—O4^i^	91.94 (5)	C1—C2—C3	116.79 (17)
O5—Co1—O4^i^	88.06 (5)	C1—C2—H2	121.6
O5^i^—Co1—O4	88.06 (5)	C3—C2—H2	121.6
O5—Co1—O4	91.94 (5)	C2—C3—C4	121.08 (18)
O5^i^—Co1—N1^i^	95.03 (5)	C2—C3—H3	119.5
O5—Co1—N1^i^	84.97 (5)	C4—C3—H3	119.5
O4^i^—Co1—N1^i^	94.77 (5)	C5—C4—C3	121.56 (18)
O4—Co1—N1^i^	85.23 (5)	C5—C4—H4	119.2
O5^i^—Co1—N1	84.97 (5)	C3—C4—H4	119.2
O5—Co1—N1	95.03 (5)	C6—C5—C4	117.63 (17)
O4^i^—Co1—N1	85.23 (5)	C6—C5—H5	121.2
O4—Co1—N1	94.77 (5)	C4—C5—H5	121.2
O2—S1—O1	115.14 (8)	C1—C6—C5	120.11 (16)
O2—S1—N1	111.29 (8)	C1—C6—C7	111.46 (14)
O1—S1—N1	110.87 (8)	C5—C6—C7	128.36 (16)
O2—S1—C1	110.64 (8)	O3—C7—N1	124.77 (15)
O1—S1—C1	110.29 (8)	O3—C7—C6	122.14 (15)
N1—S1—C1	97.19 (8)	N1—C7—C6	113.08 (14)
O4—S2—C8	104.73 (8)	S2—C8—H8A	109.5
O4—S2—C9	105.08 (8)	S2—C8—H8B	109.5
C8—S2—C9	98.91 (10)	H8A—C8—H8B	109.5
S2—O4—Co1	125.54 (7)	S2—C8—H8C	109.5
Co1—O5—H5A	108.2 (18)	H8A—C8—H8C	109.5
Co1—O5—H5B	125.2 (16)	H8B—C8—H8C	109.5
H5A—O5—H5B	108 (2)	S2—C9—H9A	109.5
C7—N1—S1	110.58 (11)	S2—C9—H9B	109.5
C7—N1—Co1	121.08 (11)	H9A—C9—H9B	109.5
S1—N1—Co1	125.03 (8)	S2—C9—H9C	109.5
C2—C1—C6	122.81 (16)	H9A—C9—H9C	109.5
C2—C1—S1	130.12 (14)	H9B—C9—H9C	109.5
C6—C1—S1	107.07 (12)		

Symmetry codes: (i) −*x*+1, −*y*+1, −*z*.

### Hydrogen-bond geometry (Å, °)

**Table:**

*D*—H···*A*	*D*—H	H···*A*	*D*···*A*	*D*—H···*A*
O5—H5A···O3^i^	0.98	1.73	2.646 (2)	155 (3)
O5—H5B···O1	0.98	2.09	2.803 (2)	128.(2)
O5—H5B···O1^ii^	0.98	2.06	2.904 (2)	143 (2)
C8—H8B···O5^iii^	0.98	2.54	3.277 (2)	132.
C8—H8B···O4^iv^	0.98	2.51	3.383 (2)	148.
C9—H9A···O3^i^	0.98	2.58	3.363 (2)	137.
C9—H9B···O4^iv^	0.98	2.58	3.432 (2)	146.

Symmetry codes: (i) −*x*+1, −*y*+1, −*z*; (ii) −*x*+1, −*y*+1, −*z*+1; (iii) −*x*+1, *y*+1/2, −*z*+1/2; (iv) *x*, −*y*+3/2, *z*+1/2.
